# Deeper Effects of fiscal multidimensional poverty reduction: household characteristics, financial lags and elite capture

**DOI:** 10.1371/journal.pone.0319255

**Published:** 2025-02-24

**Authors:** Wenjie Jiang, Hong Yang, Chunyu Liu

**Affiliations:** 1 College of Economics and Management, Xinjiang Agricultural University, Urumqi, Xinjiang, China; 2 Institute of Rural Revitalization Research, Xinjiang Agricultural University, Urumqi, Xinjiang, China; University of Innsbruck: Universitat Innsbruck, AUSTRIA

## Abstract

The governance of multidimensional relative poverty is a key challenge in rural poverty alleviation in the new era, as well as an important practice of the implementation of the United Nations Sustainable Development Goals in China. Based on provincial fiscal and financial data as well as data from the China Family Panel Studies (CFPS), this article employs multilevel linear regression and structural equation modeling to empirically examine the impact and mechanisms of fiscal investment in agriculture on multidimensional relative poverty among farmers. The research results indicate that fiscal investment in agriculture can effectively alleviate multidimensional relative poverty among rural households, and this conclusion still holds after the robustness and endogeneity tests of traditional measurement and Double Machine Learning. However, differences in household characteristics affect the performance of fiscal poverty alleviation. Households in the central and western regions, with larger family sizes, younger members, and lower levels of education, exhibit higher policy responsiveness. In terms of mechanisms, digital inclusive finance and social capital serve as important channels for fiscal multidimensional poverty reduction. However, attention should be paid to the positive lag effect of digital inclusive finance and the risk of “elite capture” in households with low levels of social capital. Accordingly, the article recommends that fiscal spending should be increased and made more efficient, with precise policy measures, strengthened institutional coordination, and efforts to cultivate optimal levels of social capital. While the article is limited by data availability to allow for a more in-depth and complex discussion, it still provides insights for fiscal strategies aimed at building high-quality shared prosperity.

## 1. Introduction

The eradication of absolute poverty was a milestone achievement of the Chinese Government within the framework of the United Nations Sustainable Development Goals. In the “post-poverty era”, the focus has shifted from eliminating absolute poverty to managing relative poverty. Relative poverty, unlike absolute poverty, emphasizes the deprivation of developmental rights, seen in disparities in basic public services, income levels, and living environments[1].China’s anti-poverty success stems from a development model under macroeconomic control, with the fiscal funds as its backbone, showcasing the strengths of the socialist system. The agricultural sector’s disadvantage in the modern industrial system makes alleviating farmers’ relative poverty and achieving common prosperity dependent on various resource integration, with the fiscal policy maintaining a leading role. In 2024, China’s agricultural, forestry, and water expenditures increased by 5.1% over the previous year. Can sustained government investment in agriculture effectively alleviate relative poverty and improve rural farmers’ sense of achievement? How do different groups perform under this policy? What are the pathways to achieving these goals? Addressing these questions forms the foundation for effective poverty governance in the new era.

Sen proposed a framework for analyzing multidimensional poverty based on relative deprivation of rights, and since then research has focused on the study of multidimensional poverty measurement methods and the analysis of governance measures [2]. Education, healthcare, and water are important components of multidimensional poverty, while spatial and geographic factors have a significant impact on poverty levels, implying that the role of government should be emphasized in the governance of multidimensional poverty [3]. A well-governed government is effective in alleviating multidimensional poverty in non-low-income countries [4], while evidence from Bangladesh suggests that in low-income countries, the integration of microfinance and government programs, as well as NGO programs, can also alleviate inequality [5]. In China, the role of government is closely linked to the formulation of fiscal policy and the use of fiscal resources. The academic consensus is that fiscal expenditure plays a crucial role in revenue generation, supporting China’s continued developmental poverty alleviation efforts[6]. The role of fiscal policy in governing relative poverty has been empirically validated. Transfer payments, as a source of local revenue, create employment opportunities and reduce local relative poverty[7]. From the perspective of expenditure, the productive expenditure of the fiscal strengthens the level of infrastructure construction, promotes the development of rural and agricultural development, and then alleviates relative poverty. Fiscal productive expenditures can also promote economic development, regulate the internal distribution of rural areas, and provide an institutional guarantee for rural poverty reduction [8]. Poverty has the characteristic of intergenerational transmission, and financial expenditure on education can effectively reduce the probability of intergenerational transmission of multidimensional relative poverty in rural areas, especially for low- and middle-income families the effect is more significant[9]. International practice shows that public policy interventions are equally significant in improving multidimensional poverty. Studies in Mozambique, Brazil, and Uganda illustrate that government public policies can achieve multidimensional poverty alleviation through the provision of public health services, drought response, and personal finance training [10–12].However, some argue that fiscal spending does not positively affect income distribution among farmers or alleviate relative poverty. For example, agricultural subsidies can reduce absolute poverty and increase low-income farmers’ earnings but do not alter income distribution patterns [6]. To stabilize the economy, local governments have implemented tax cuts and fee reductions, which has increased fiscal pressure. In this context, fiscal pressure has worsened the relative poverty of farmers[13]. From a horizontal equity perspective, poverty reduction policy inputs are derived from taxes, and while poverty reduction policies alleviate poverty for some people, they also make the marginalized or non-poor population of the poverty standard poor [14]. In addition to the above reasons, labor market access, risk protection, financial supply and supply-side constraints likewise make the mitigating effect of government fiscal inputs on multidimensional poverty constrained [15]. In summary, although the practice of the Chinese government illustrates the effective role of fiscal inputs on multidimensional relative poverty, academic differences still need to be bridged, and for this reason, one of the main objectives of the article is to verify the existence of the role of the Chinese government’s fiscal inputs on the mitigation of multidimensional relative poverty and to further consider how the fiscal can play a role. As the fundamental unit of agricultural production and risk-taking, household characteristics significantly impact policy implementation performance. Region, education, population, and age are key factors in household characteristics. Regional differences are crucial for understanding farmers’ behavior. China’s regional economic development shows significant disparities, influencing farmers’ livelihood choices. This results in a market-driven family livelihood model in the east, and a government-driven intergenerational labor model in the central and western regions[16]. Differential livelihood patterns create differences in the level of individual response to policy implementation. Education strongly influences individual cognition, which in turn affects the livelihood strategies chosen by farming households[17]. Households with higher education levels are generally believed to alleviate multidimensional relative poverty by expanding social participation and non-farm employment, while also preventing its intergenerational transmission[9,18]. However, education, as a household investment, carries costs and risks. For low-income households, education expenses are a heavy burden and may not effectively reduce poverty[19]. Household labor force participation is a key indicator of development potential and reflects household endowments. Households with a large labor force and a low average age exhibit higher participation rates and better poverty reduction outcomes [20]. In the “post-poverty era,” achieving high-quality poverty governance requires further clarification on where and how to allocate poverty alleviation resources.

In this context, digital inclusive finance, as the primary policy-based financial tool, can effectively alleviates multidimensional relative poverty through government fiscal resource support. Digital inclusive finance reduces farmers’ financial constraints and fosters innovation, then helping manage their relative poverty [21]. However, the digital divide and differences in financial literacy contribute to the “Matthew effect”, where the poverty reduction impact on the extremely poor remains limited [22]. In some developing or underdeveloped countries, there is still a mismatch between the supply and demand structure of inclusive finance, and there is still a discrepancy between formal financing channels and the sustained and inclusive supply needed by rural smallholder farmers [23]. The role of financial inclusion is simultaneously influenced by unconventional factors, such as religion. Research on financial inclusion in Islamic countries suggests that banking development and financial inclusion factors have not improved income inequality and poverty eradication in Islamic countries [24].Social capital represents informal social institutions, which can serve as a substitute for formal institutions to some extent during the implementation process. At the same time, it can also work synergistically with formal institutions [25,26]. Social capital is important for reducing rural poverty and is strongly associated with the level of household poverty [27,28]. From the experience of developing countries, social capital has a mitigating effect on both monetary and multidimensional poverty [29]. At the same time, social capital shows strong “pro-poorness”, i.e., the lower the income level, the greater the impact of social capital on poverty reduction [30]. Acquaintance society is a typical characteristic of rural China. Under the impact of marketization, with the gradual disintegration of close-knit rural communities and the rise of elite governance, interpersonal relationships within these familiar communities can be embedded into public governance. This embedding can help consolidate consensus among stakeholders, stimulate individual participation, reshape village public life, and further alleviate relative poverty[31]. Clans based on kinship ties remain a crucial part of contemporary rural life. Clans serve as key carriers of social capital for Chinese farmers, with clan participation enhancing social support and reducing farmers’ relative poverty both socially and economically[32]. However, clans pose theoretical risks to common prosperity, including community exclusion and elite capture[33]. The non-linear role of social capital is not unique to China. An interview study of a Dutch community demonstrated that farmers’ social networks in poor areas are small and fragmented, which corresponds to the unbalanced and powerful position of community leaders, which has the potential to exacerbate social exclusion [34]. At the same time, differences in the structure of social capital as well as too high and too low levels of social capital may be associated with high rates of poverty in the community [35].

In summary, existing studies have developed a wealth of findings around fiscal poverty reduction and its mechanisms, affirming the role of financial poverty reduction, recognising the impact of household characteristics on poverty reduction performance, and recognising the role of digital financial inclusion and social capital as mechanisms. However, there are still some shortcomings in the existing studies. First, The role of public fiscal funds in multidimensional poverty reduction has not yet been harmonized and agreed upon in the context of national governance experiences;; Second, There is no unified understanding of the governance of relative poverty characterized by intra-farmer developmental disparities, especially in educational interventions; Third, there is a lack of discussion on the governance performance of digital financial inclusion in the time dimension; Fourthly, there is a lack of empirical evidence on the risk of “elite capture” in the development of social capital.

The shift in fiscal policy from “flood irrigation” to “precision drip irrigation” requires an analysis of household characteristics in order to implement “targeted measures.” The profit-seeking nature of financial capital and the fragmentation of traditional social capital make it difficult for them to independently address relative poverty. Therefore, it is particularly important to discuss which factors can facilitate the orderly infusion of financial capital into rural areas and the expansion of rural social capital resources.This article, based on provincial fiscal data, digital inclusive finance, and 10010 household data from the China Family Panel Studies (2014–2022), discusses the poverty reduction performance of fiscal support for agriculture in relation to farmers’ relative poverty. It further analyzes the path mechanisms between fiscal support for agriculture, digital inclusive finance, social capital, and relative poverty.

The marginal contributions of this article are as follows: First, in developing countries, poverty governance requires the government to invest, guide and underwrite, so as to drive all parties to invest in the process of poverty governance. In the face of the divergence in the performance of fiscal poverty governance, based on macro-nested micro-data, the group heterogeneity of policy performance is further discussed on the basis of fiscal poverty reduction, and the positive roles of governmental public policies and fiscal funds are clarified, so as to provide empirical reference for developing countries and even the global poverty governance and the realization of the United Nations sustainable development goals. Second, While existing studies have affirmed the role of financial inclusive development on poverty governance and recognized that financial inclusion still lacks a role for the very poor individuals, the article further proposes and validates the lag effect of digital inclusive finance, indicating that it may have negative impacts on poverty governance in the short term but positive effects in the long term. Third, On the basis of affirming that social capital promotes equality and inhibits poverty, the article empirically analyzes the existing facts and possible historical economic factors of the non-linear impact of social capital on poverty governance, revealing that the phenomenon of ‘elite capture’ may result when social capital is either low or too high, which provides new perspectives on poverty governance in regions where governmental power is inadequately embedded Provides new perspectives on poverty governance in areas where government power is insufficiently embedded, such as rural areas in China with a high degree of clannishness or rural areas in less developed countries with a high degree of influence from non-government authority.

## 2. Theoretical analysis and research hypotheses

### 2.1. Impact of fiscal support for agriculture on relative poverty alleviation and differences in their household characteristics

According to Peter Townsend, relative poverty refers to the relative deprivation of a group in terms of resources, power, opportunities, etc., when compared with an established reference group. Combined with the context of building common prosperity in rural China, relative poverty can be understood as the unequal distribution of income within farmers and inadequate access to basic public services. Taking “Agriculture, Forestry and Water Resources Expenditure” in the “Classification of Government Revenue and Expenditure in 2024” as an example, the use of fiscal support for agriculture can be broadly classified into four categories: Firstly, organisational construction, such as the expenditure of administrative institutions and the construction of collective economic organisations; Secondly, inputs into public services, such as roads, flood control and disaster prevention, and education; Thirdly, bottom-up protection, such as the provision of income subsidies, etc. Fourthly, industrial support, such as loan subsidies.

In terms of direct effects, fiscal support for agriculture has helped to alleviate relative poverty in terms of raising efficiency and increasing income and regulating the structure of income distribution. Through the construction of roads, water and electricity, communications and other infrastructure and investment in education, health care and other social services, fiscal funds have achieved industrial capital agglomeration and human capital accumulation, suppressed the inequality of public services in rural areas, and improved the efficiency of agricultural production[36]. At the same time, the accumulation of current human capital can reduce the viscosity of intergenerational transmission of human capital in low-income groups and prevent the intergenerational transmission of relative poverty[37]. In addition, the input of fiscal funds of the nature of the pocket guarantee is of the nature of “benefiting poverty”, which is conducive to narrowing the income distribution gap, alleviating the income constraints of the low-income groups, and contributing to the accumulation of livelihood capital [38].

The performance of fiscal poverty reduction is further analysed in detail by combining the differences in household characteristics. There is spatial variability in the efficiency of China’s fiscal support for agriculture[39], and due to the unevenness of China’s regional development, different cultural and industrial backgrounds in different geographical areas make differences in the livelihood strategies of farming households in different places[38], so in the context of regional differences in both the supply of and the demand for fiscal poverty alleviation, the policy response of farming households to the fiscal support for agriculture varies from place to place. Household size, household age, and education level are important household endowments, reflecting household social participation, risk preference and sharing, and innovation awareness. Large household size implies, to some extent, a large labour force and low income constraints, on the basis of which household members have higher risk appetite for expanding their business or entrepreneurship, and have better levels of risk sharing and risk income[40]. Households also have a life cycle, and the degree of ageing affects a household’s business strategy and economic risk[41]. Higher levels of ageing make households more risk averse[42], which makes them more conservative and less able to respond flexibly to external policy shocks, so that larger, younger households are better able to capture the positive spillover effects of policy shocks. The lack of education is the lack of development potential of the household. Undereducated farm households tend to have inadequate cognitive levels, which in turn constrains their behavioural choices and livelihood strategies such as business and entrepreneurship[17]. The existence of cognitive differences also affects the level of response of farm households to policies, thus low education level farm households are unresponsive and dependent on policies. Therefore, in the multidimensional relative poverty rule, fiscal expenditure has better performance for low education level households due to its inclusiveness instead[43]. To this end, the article proposes the following hypothesis:

**H1a:** Fiscal support for agriculture can effectively alleviate the relative poverty of farm households.

**H1b:** Fiscal support policies for agriculture have different performance for households with different characteristics.

### 2.2. Mechanisms of fiscal support for agriculture to alleviate multidimensional relative poverty

The theory of “new endogenous development” considers that rural areas are both locally rooted and interactively orientated towards the outside world[44].Therefore, the alleviation of rural relative poverty requires the effective integration of internal and external resources. Digital inclusive finance and social capital, as representatives of external and endogenous resources, formal and informal systems, respectively, can achieve the adjustment of income distribution and the acquisition of the right to development of peasant groups in order to alleviate relative poverty.

Digital financial inclusion represents financial capital to the countryside, can effectively overcome financial exclusion, and was once seen as an effective tool for income generation and poverty governance[45].Digital financial inclusion can alleviate the financial constraints of rural households, reduce financing costs, and thus narrow the income gap. However, it should also be noted that the common wealth effect of digital inclusive finance is more pronounced in regions with a wide distribution of financial institutions and high levels of education and Internet penetration[46]. The “Matthew effect” effect of financial inclusion in the countryside is still evident among rural households with low solvency and low financial market participation[47].The article focuses on relative poverty and equality of basic livelihood rights and income distribution among rural households. In the face of the lower level of development of rural households, the financial capital gap still exists, and farmers still face information asymmetry, insufficient collateral, and difficulty in supporting knowledge and human capital when accessing borrowing capital. At the same time, relative poverty is the result of multiple attributions, such as institutions and social services, and it is difficult for financial capital to address the complex root causes. As the financial capital divide is bridged, financial penetration is increased, widely disseminated among different user groups, and financial instruments will gradually play a positive role as the overall knowledge accumulation and human capital of the farming community is enriched, which will take time. Therefore, the article proposes the following research hypothesis:

**H2a:** Digital financial inclusion does not have a positive effect on relative poverty governance in the current period;

**H2b:** Digital financial inclusion has a time lag in relative poverty governance.

As an informal institution, social capital has the potential power to broaden the path to shared prosperity when nested within the formal system. Social capital is externally characterised by social networks, social trust and social norms. Studies have affirmed the role of public policy in fostering social capital [48]. Public fiscal investment in rural public infrastructure enriches social capital by expanding farmers’ social networks and sources of information. For example, the internet infrastructure constitutes the main carrier of social capital, expanding the social dimension of farmers, extending social networks, strengthening social trust, and promoting social norms[49].Rural organisational development is also the basis for the cultivation of social capital. Under the support of fiscal resources, strengthening the construction of rural grassroots party organisations, giving full play to the effectiveness of the governance of grassroots organisations, supporting the cultivation and development of collective economic organisations, and integrating individual farmers into the collective economic organisations are effective measures for expanding the social network of farmers. The cultivation of social capital will ultimately break down the “the order of stratified closeness” in the countryside. Based on social networks, farmers can transfer and obtain technology, information, capital and other factors, which can help production, expand employment channels and stimulate entrepreneurial practice; social networks also constitute social security for farmers, providing informal risk avoidance. Based on social trust and social norms, and fostering the spirit of contract, farmers are better embedded in the market economy, while lowering transaction and institutional costs, and providing a guarantee for poverty reduction and distributional adjustment. In addition, it is important to note that social capital also acts as a disincentive to shared prosperity[33]. When the overall level of social capital is low, it means that social capital is over-concentrated and excluded within a small community, and they can use social networks to monopolise the flow of information and control the allocation of resources. These people have access to public resources through personal relationships or specific social networks, and the process of resource allocation is less transparent, making it difficult for ordinary members to have equal access. For example, in rural areas, “capable people ruling the village” or family power may be better than ordinary villagers in accessing policy resources; inter-regional, intercultural, and even cross-class competition may activate the negative effects of social capital. Therefore, the article puts forward the following hypotheses:

**H3a:** Fiscal support to agriculture can achieve relative poverty alleviation by increasing the level of social capital;

**H3b:** The alleviation of relative poverty by social capital is non-linear.

**Fig 1:**
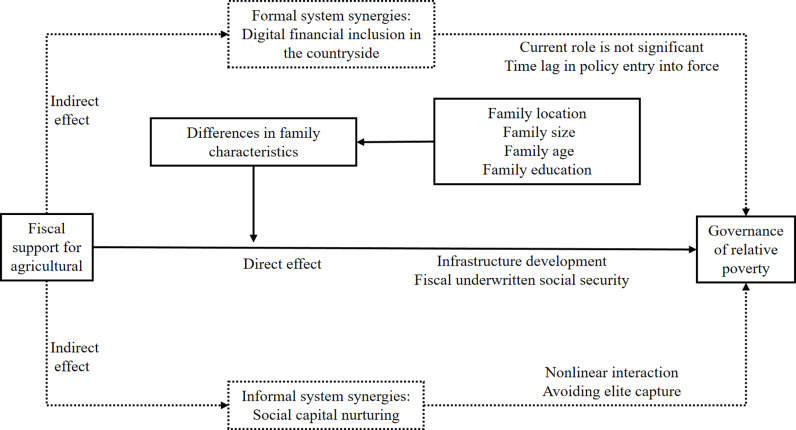
Theoretical analysis framework.

In summary, the theoretical framework of the role of fiscal support for agriculture in the generation of multidimensional relative poverty of farm households is formed, as shown in Fig 1.

## 3. Empirical research design

### 3.1. Data sources

The main data comes from the China Family Survey (CFPS) data released by the China Social Science Survey Centre of Peking University, which is conducted once every 2 years and adopts a random sampling method to collect data on urban and rural residents’ household income, production, etc., with good national and provincial representativeness, and which is currently updated until 2022. Given that the article’s research needs to be aligned with provincial urban-rural macro data, the urban-rural attributes of the research sample are judged by the standards released by the National Bureau of Statistics. The article combines the adult pool, the household pool, and the juvenile proxy pool to form the cross-section data at the level of agricultural households and combines the cross-section data for the years 2014, 2016, 2018, 2020, and 2022 into the panel data, eliminating missing values and invalid samples, and ultimately retaining 10010 samples for the 26 provincial-level administrative districts. The fiscal support for agriculture and other data are taken from China Statistical Yearbook (2015–2023) and China Rural Statistical Yearbook (2015–2023), and the digital financial inclusion index is taken from the Digital Financial Inclusion Index released by the Digital Finance Centre of Peking University, which officially releases data for the years of 2011–2020 only. The data for 2022 used in the succeeding article relies on the publicly available methods and indicators of this group, and is supplemented by its own calculation based on the availability of data [50]. The data are nested because they are matched from the macro-financial data to the micro-household level.

### 3.2. Variable selection and measurement

Explained variables: multidimensional relative poverty index (*Mpscor*) and multidimensional relative poverty status (*Mpi*). The mainstream international measurement of multidimensional relative poverty is based on the Multidimensional Relative Poverty Index (MPI) developed by the United Nations Development Programme (UNDP) and the Oxford Poverty and Human Development Initiative (OPHI), which consists of the three dimensions of health, education and living standards. Most of the existing studies follow this standard, or make moderate adjustments in the selection of indicators based on the object of study. For example, in order to discuss multidimensional poverty in India after Covid-19, researchers constructed the same index dimensions of health, education and living standards, but there are differences in the specific indicators [51]. Research on multidimensional relative poverty advocates going beyond mere income or consumption poverty [52], but it has to be admitted that earning capacity is still an intuitive indicator reflecting the state of poverty. In China’s precision poverty alleviation practice, the criteria for poverty eradication revolve around “One Income, Two Guarantees and Three Safeguards”. For this reason, based on the theory of viability and the MPI, and with reference to the existing research at[53], we have formed an evaluation system consisting of 12 indicators in 5 dimensions, including income, social security, living standards, education and the economy, as shown in Table 1.

**Table 1:** Indicator system for multidimensional relative poverty.

Dimensions	Indicators	Deprivation threshold definition
Incomes (1/5)	Income gap (1/12)	Household per capita income below 50 percent of the sample median is 1
Social security (1/5)	Self-assessed health status (1/12)	Any family member rated himself/herself as unhealthy, with a child proxy answer of 1 for health status less than or equal to 3
	Medical protection (1/12)	The presence of one or more persons in the household without any medical coverage is assigned a value of 1.
	Old-age protection (1/12)	Old-age insurance coverage for adults in the household is below the sample median assigned a value of 1
Education (1/5)	Youth out of school (1/12)	Adolescents aged 7–15 are out of school with a value of 1.
	Educational attainment (1/12)	Highest average household education level below the median for the same year assigned a value of 1
Living standards (1/5)	Drinking water (1/12)	Unsafe water use for non-well water, tap water and barrel water assigned a value of 1
	The internet (1/12)	Households’ average awareness of the Internet is below the median of 1, i.e., a score of 1–2
	Domestic fuel (1/12)	Non-liquefied, natural, solar, electric and other clean fuels are assigned a value of 1
Economics (1/5)	Consumption of durable goods (1/12)	Consumption of durable goods at or below the median is assigned a value of 1
	Presence of unemployment (1/12)	The existence of unemployment in the labour force is assigned a value of 1
	Whether or not you own your own home (1/12)	No is assigned to 1

Based on the selection of the above indicators, the Alkire-Foster (A-F) method was used to determine whether individual family farmers were in multidimensional relative poverty and to calculate their multidimensional relative poverty index. The basic calculation logic is shown in equations (1)-(3). Equation (1) calculates the poverty index that indicates whether household *i* is poor on dimension *d* (yes =  1), and refers to the weight of the dimension, usually a poverty threshold is set 0.3, when the poverty index is lower than 0.3, it means that the household is in multidimensional relative poverty; Equation (2) calculates the incidence of poverty and the depth of poverty, the depth of poverty refers to how many dimensions a household in multidimensional relative poverty is poor in on average; And further calculates the multidimensional relative poverty index from equation (3).


$$c_{i}=\sum_{d=1}^{12}w_{d}I_{id},w_{d}=\frac{1}{12}$$ (1)

$$H=\frac{q}{n},A=\frac{1}{q}\sum_{i=1}^{q}\frac{c_{i}}{12}$$ (2)

Mpiscor=H×A (3)

Explanatory variables: Fiscal support for agriculture (*Finrate*). Expenditure on agriculture, forestry and water can reflect the government’s support for the “three rural areas”, but the fiscal strength of each province is different, so the proportion of expenditure on agriculture, forestry and water in the general public budget expenditure as a proxy variable.

Control variables: Based on the availability of data from the China Family Panel Studies (CFPS) and drawing on existing research[54], control variables were selected from the levels of household head, household, and region. At the household head level, the “family financial respondent” was designated as the household head, and nine control variables were selected: gender of the household head (*Gender*), age of the household head (*Age*), whether the household head is a party member (*Party*), years of education of the household head (*Edu*), marital status of the household head (*Marriage*), health condition of the household head (*Health*), religious beliefs of the household head (*Religion*), internet literacy of the household head (*Internet*), and interpersonal relationships of the household head (*Relation*). At the household level, the household population size (*Familysize*) and the nature of the residence (*Residence*) were chosen as control variables. At the regional level, the logarithm of the regional economic development level (*Lgdp*) was selected as a control variable.

Other variables: In order to achieve the mediation effect analysis, the logarithm of the digital inclusive finance index released by the Digital Finance Centre of Peking University (*Lindex*) is used as the mediator variable; Drawing on the results of the existing results, the logarithm of the “expenditure on favours and gifts” is used as a proxy for the social capital (*Lsocial*) variable[54]. In addition, for the robustness test, the logarithm of per capita agriculture, forestry and water expenditures (*Lperfin*) is used as an alternative explanatory variable; In order to alleviate the endogeneity problem due to sample selection, mutual causality, etc., the proportion of agriculture, forestry and water expenditures in general public budget expenditures in the previous year *(Prefinrate)* and the area of disaster in the province (*Damage*) are selected as instrumental variables to conduct the endogeneity test, respectively. The results of descriptive statistics of variables are shown in Table 2.

**Table 2:** Descriptive statistics of variables.

Variable	Description of variables	Obs	Mean	Std. Dev.	Min	Max
*Mpi*	“1” for multidimensional relative deprivation	10010	0.290	0.454	0	1
*Mpiscor*	Multidimensional relative poverty index	10010	0.229	0.147	0	0.700
*Finrate*	Share of agriculture, forestry and water Expenditures in general public budget expenditures	10010	0.121	0.033	0.040	0.190
*Lsocial*	Logarithmic expenditure on favours	10010	7.141	2.216	0	11.695
*Lindex*	Digital financial inclusion index takes logarithms	10010	5.583	0.284	5.041	6.116
*Lperfin*	Logarithm of per capita expenditure on agriculture, forestry and water	10010	8.057	0.409	7.155	9.745
*Damage*	Area affected by a disaster	10010	400.966	341.047	0	2664
*Prefinrate*	Share of expenditure on agriculture, forestry and water in general public budget expenditure in the previous year	10010	0.121	0.031	0.045	0.183
*Gender*	Sex of head of household, male “1”	10010	0.590	0.492	0	1
*Age*	Age of head of household	10010	52.457	12.484	15	90
*Party*	Whether the head of household is a member of the Communist Party	10010	0.095	0.293	0	1
*Edu*	Years of education of the head of household	10010	6.169	4.297	0	19
*Marriage*	Is the head of the household in the marriage	10010	0.887	0.316	0	1
*Health*	Health status of the head of household	10010	0.678	0.467	0	1
*Religion*	Whether the head of the household is religious	10010	0.392	0.488	0	1
*Internet*	Internet Literacy for Household Heads	10010	0.352	0.477	0	1
*Relation*	Self-assessment of interpersonal Relationships by the head of household	10010	0.771	0.420	0	1
*Familysize*	Household size	10010	4.126	1.948	1	15
*Residence*	Household residence, take “1” for rural areas.	10010	0.207	0.405	0	1
*Lgdp*	Gross regional product in logarithms	10010	10.129	0.753	8.827	11.768

### 3.3. Modelling

The article selects provincial nested household data to discuss the causal relationship between fiscal support for agriculture and multidimensional relative poverty. The multilevel linear regression model can better account for the hierarchical structure of the data, separate individual and group effects, and provide more precise results. The model allows for different starting levels of the dependent variable in different provinces and different magnitudes of the effect of the independent variable on the dependent variable between provinces, so the random intercept and random slope are set. In addition, the model considers clustering of standard errors at the provincial level. And the model is as follows:


$$Mpiscor_{ij}=\beta_{0}+\beta_{1}Finrate_{ij}+\beta_{2}\sum Control_{ij}+i.year+u_{0j}+u_{1j}Finrate_{ij}+\varepsilon_{ij}$$ (4)

In the model, in addition to the dependent and independent variables, the are control variables, the i.year are fixed year effects, and is the provincial random intercept, indicating differences in starting relative poverty levels across provinces, the finrat is the provincial random slope, indicating the varying strength of the effect of financial support to agriculture on multidimensional relative poverty across provinces, and is the residual term.

In order to discuss the mechanism role of fiscal support to agriculture on multidimensional relative poverty, it is more appropriate to use structural equations for the estimation of the mediating effect, taking into account the hierarchical structure characteristics of the data, and the model is estimated with the inclusion of the random effect of provinces, which indicates the different impacts of provinces on this effect. The model is set up as follows:


$$Mediation_{ij}=\chi_{0}+\chi_{1}Finrate_{ij}+\chi_{2}\sum Control_{ij}+u_{1}$$ (5)

$$Mpiscor_{ij}=\lambda_{0}+\lambda_{1}Finrate_{ij}+\lambda_{2}Mediation_{ij}+\lambda_{3}\sum Control_{ij}+u_{2}$$ (6)

In the model, the mediatio denote the mediating variables digital financial inclusion index (*Lindex)* and social capital (*Lsocial*), respectively.u are the residual terms. To discuss the time lag effect of digital financial inclusion, a dynamic panel model is constructed; to discuss the nonlinear effect of social capital, the primary and secondary terms of social capital are included in a multilevel linear regression model, respectively:


$$\begin{array}{l} Mpiscor_{ij}=\eta_{0}+\eta_{1}Lindex_{ij}+\eta_{2}Lindex\_lag_{ij}+\eta_{3}Finrate_{ij}\\ +\eta_{4}\sum Control_{ij}+u_{0j}+u_{1j}Finrate_{ij}+\varepsilon_{ij} \end{array}$$ (7)

$$\begin{array}{l} Mpiscor_{ij}=\theta_{0}+\theta_{1}Finrate_{ij}+\theta_{2}Lsocial_{ij}+\theta_{3}Lsocial^{2}\\ +\theta_{4}\sum Control_{ij}+i.year+u_{0j}+u_{1j}Finrate_{ij}+\varepsilon_{ij} \end{array}$$ (8)

## 4. Analysis of measurement results

### 4.1. Baseline regression results

The baseline regression of fiscal support to agriculture on multidimensional relative poverty is shown in Table 3. Models 1–4 represent the results of not adding control variables, adding control variables at the head of household level, household level, and district level, respectively. The regression coefficient of fiscal support for agriculture in model 1 is -0.920, which is significant at the 95% confidence level, and the control variables at each level are added one by one to model 4, and the coefficient is -0.734, which is still significant at the 95% confidence level, and the model is more robust. The regression results indicate that the estimated coefficient of fiscal support for agriculture is significantly negative regardless of whether the control variables are added or not, indicating that fiscal support for agriculture can effectively manage the multidimensional relative poverty of farm households, which verifies the conclusion of hypothesis H1a. With the continuous “blood transfusion” of public fiscal resources, the rural economy and farmers’ welfare have been greatly improved, and the relative poverty of farmers has been alleviated by the “trickle-down effect” in the context of overall rural development. In addition, inclusive and pro-poor fiscal spending has raised the level of rural public infrastructure and improved rural governance capacity, which has ensured the development of rural industries, poverty reduction and prosperity for farmers, and distributional equity.

**Table 3:** Benchmark regression results.

Variable	Model 1	Model 2	Model 3	Model 4
***Finrate***	-0.920*** (0.326)	-0.772** (0.287)	-0.774*** (0.283)	-0.734*** (0.276)
***Gender***		0.0153*** (0.004)	0.015*** (0.004)	0.016*** (0.004)
***Age***		0.002*** (0.001)	0.002*** (0.001)	0.002*** (0.001)
***Party***		-0.013 * (0.006)	-0.013** (0.006)	-0.013** (0.006)
***Edu***		0.009*** (0.001)	0.009*** (0.001)	0.009*** (0.001)
***Marriage***		-0.020*** (0.005)	-0.013*** (0.005)	-0.013*** (0.005)
***Health***		-0.039*** (0.005)	-0.039*** (0.005)	-0.039*** (0.005)
***Religion***		-0.010*** (0.003)	-0.009*** (0.003)	-0.009*** (0.003)
***Internet***		-0.045*** (0.003)	-0.046*** (0.003)	-0.046*** (0.003)
***Relation***		-0.025*** (0.008)	-0.024*** (0.008)	-0.024*** (0.008)
***Familysize***			-0.006*** (0.002)	-0.006*** (0.002)
***Residence***			-0.011** (0.005)	-0.011** (0.005)
***Lgdp***				-0.027*** (0.010)
***Constant***	0.341*** (0.000)	0.346*** (0.048)	0.374*** (0.047)	0.645*** (0.139)
***Time***	Yes	Yes	Yes	Yes
***Province***	Yes	Yes	Yes	Yes
***Obs***	10010	10010	10010	10010

Note:

* p < 0.05,

** p < 0.01,

*** p < 0.001. Standard errors are provincial clustering standard errors. If not otherwise specified, the same below.

In terms of control variables, the regression coefficients for both gender and age are significantly positive, meaning that the multidimensional relative poverty of households tends to deepen when the head of the household is male and as the head of the household grows older. The reason for gender differences in poverty may lie in pro-poor policy preferences. Academics and practitioners have long recognised the disadvantages of rural women in terms of income and poverty[55].Therefore, they have paid attention to the protection of women’s rights in all aspects of poverty alleviation and development, such as supporting “women’s poverty alleviation workshops” and women’s health screening, etc. Therefore, the empirical results show that male-headed households are more prone to fall into multidimensional relative poverty due to the gender difference and policy orientation. The increase in the age of the head of the household implies, on the one hand, that the labour capacity of the head of the rural household is weakening, which means that his or her support for the household is decreasing; on the other hand, it implies that the degree of ageing of the household is increasing, which means that it is more likely to fall into multidimensional relative poverty.

### 4.2. Robustness and endogeneity tests

In order to verify the robustness and reliability of the estimated coefficients and conclusions, on the basis of the baseline regression adding control variables one by one, the method of replacing the explanatory variables and replacing the core variables at the same time is used to conduct the robustness test. Replace the original explanatory variables with whether the state of multidimensional relative poverty (*Mpi*), because *Mpi* is a 0/1 variable, and take logit regression, the results are as in model 5. Unlike the interpretation of the economic significance of the linear model, the logit model economic significance is a reflection of probability. The coefficient is -8.307, which is significantly negative, implying that as fiscal support for agriculture increases, the log odds of a farm household falling into multidimensional relative poverty will significantly decrease. There is a negative relationship between the two. Model 5, which simultaneously reports the marginal effects of the independent variables computed at the mean points of all variables in the *Odd* column, suggests that the probability of a farm household falling into multidimensional poverty will decrease by 1.485 percent as the unit level of fiscal support for agriculture increases. Further replace the independent variables and dependent variables at the same time, and take the logarithm of per capita expenditure on agriculture, forestry and water (*Lperfin*) as the independent variable, and the results are as in Model 6,the regression coefficient is -0.645, which is significantly negative, indicating that for every unit increase in the logarithm of per capita expenditure on agriculture, forestry and water, the log odds of multidimensional poverty for farm households will decrease by 0.645, verifying the negative relationship between fiscal support for agriculture inputs and multidimensional poverty for farm households. The marginal effect is reported in the *Odd* column of Model 6 and is negative at the 10% significance level with a coefficient of -0.125, which means that for every unit increase in fiscal support for agriculture measured by the log of per capita expenditure on agriculture, forestry and water, the probability of a farm household falling into multidimensional relative poverty will fall by 0.125 percentage points. The results of the robustness test are in line with those in the benchmark regression, and it confirms that the fiscal support to agriculture can effectively alleviate the multidimensional relative poverty of farm households.

Further endogeneity test is conducted with the area of disaster (*Damage*) and the share of agriculture, forestry and water expenditure in general public budget expenditure in the previous year (*Prefinrate*) as instrumental variables and using two-stage regression in an attempt to alleviate the endogeneity problem caused by omitted variables, sample selection, mutual causation, etc. It is usually believed that natural phenomena are characterized by a high degree of stochasticity and exogeneity, which are not related to individual heterogeneity but can affect the socio-economic system, so the natural environment has become the mainstream instrumental variable selection idea [56]. The area of disaster refers to the area deeply affected by natural disasters, after the disaster, the local government will invest a lot of money to restore the land, and the size of the land resilience is also closely related to the government’s investment in land resilience in the previous period, so the area of disaster has a high correlation with the fiscal support for agriculture. At the same time, the area of disaster is less relevant to the multidimensional poverty that focuses on the disenfranchisement of farm households. Fiscal investment in a particular area tends to be sustainable, especially in the context of the Chinese government’s strong emphasis on agricultural production. Therefore, government spending on agriculture in the previous year has a significant impact on the current year, while poverty in the current period does not have an impact on fiscal investment in previous periods. Therefore, using the disaster area and past fiscal data as instrumental variables can “cut off” the possible correlation between the independent variable and the error term, and also “cut off” the possible mutual causation between the independent variable and the dependent variable. In addition, through two-stage regression, the instrumental variables form a prediction of fiscal inputs in the first stage, eliminating the interference of omitted variables, and forming an unbiased estimate in the second stage. For this reason, the problems of omitted variables and two-way causation, which are highly susceptible to endogenous bias, are mitigated, as seen in Models 7 and 8.In the IV-2SLS regression results, the LM and F-tests indicate that the selected instrumental variables pass the under-identification test and the weak instrumental variable test, proving that the instrumental variables are significantly correlated and valid with the endogenous variables. The coefficients of the independent variables are significant at -1.870 and -0.226 respectively. This result reinforces the validity of the above conclusion and hypothesis H1a.

Further, compared with traditional econometric models, Double Machine Learning (DML) is able to deal with the “curse of dimensionality” and multicollinearity problems that may exist in high-dimensional control variables. It can automatically identify possible nonlinear relationships in the model and alleviate the problems of model setup bias, endogeneity and omitted variables through algorithms and nonparametric modeling. In addition, DML has a two-stage process. In the first stage, the residuals are formed by predicting the control and independent variables to remove the endogeneity of the variables, and then the predictions are formed in the second stage to form a “clean” unbiased estimate. At the same time, DML reduces overfitting through cross-validation and improves predictive power and robustness. To this end, a partially linear Double Machine Learning model is used as a supplement to the robustness and endogeneity tests, and the model setup is shown in equation (9)-(10), and the auxiliary regression is constructed to accelerate the convergence, as shown in equation (11)-(12). The number of folding is set to 5, and the individual effect, time effect, and year and province interaction term are further controlled on the basis of control variables, and the causal relationship between fiscal investment in agriculture and multidimensional relative poverty of farm households is verified by neural network algorithm, and the results are shown in Table 4, Model 9, and the prediction coefficients are significantly positive at 0.1% level of significance, the coefficient is -1.007 and its main conclusions are consistent with the results of the traditional measure.

**Table 4:** Robustness and endogeneity test results.

Variable	Model 5		Model 6		Model 7	Model 8	Model 9
	*Mpi*	*Odd*	*Mpi*	*Odd*	*Iv=Damage*	*Iv=Prefinrate*	*Mpiscor*
***Finrate***	-8.307 ^*^ (3.623)	-1.485 ^*^ (0.664)			-1.870 ^*^ (0.721)	-0.226^**^ (0.067)	-1.007^***^ (0.113)
***Lperfin***			-0.645 ^*^ (0.317)	-0.125 ^*^ (0.066)			
***Constant***	4.495 ^*^ (2.076)		7.727^***^ (3.687)		1.235^***^ (0.319)	0.511^***^ (0.035)	
***Control***	Yes	Yes	Yes	Yes	Yes	Yes	Yes
***Obs***	10010	10010	10010	10010	10010	10010	10010
***Anderson LM***					77.173^***^	8648.444^***^	
***Wald F***					777.664	5.5e + 04	


$$Mpiscor_{it}=\alpha_{0}Finrate_{it}+g(Control_{it})+U_{it}$$ (9)

$$E(U_{it}|Finrate_{it},X_{it})=0$$ (10)

$$Finrate_{i,t}=m(X_{i,t})+V_{i,t}$$ (11)

$$E(V_{i,t}|X_{i,t})=0$$ (12)

### 4.3. Analysis of differences in household characteristics in poverty reduction performance

In order to further discuss the differences in the poverty reduction performance of fiscal investment in agriculture on multidimensional relative poverty among different households, the analysis is carried out in terms of the spatial location of households and household endowment. China’s economic development gradient gap is obvious, the coastal region has a high level of economic development, rural service facilities, farmers’ livelihood capital and livelihood strategies are significantly different from those in the central and western regions, so the variable *Zone* is set to assign a value of 1 to the eastern coastal provinces, and 0 to the rest of the provinces for the discussion of the sub-region. Considering the human capital accumulation from household endowment, it has both quantitative and qualitative connotations, and in general, the quantitative dividend of human capital works better in the period of low level development, and then transforms into the driving role of qualitative dividend. The average household size of rural households in the sample is 4.154, so we take 4 as the critical value, and discuss the poverty reduction performance of fiscal support for agriculture on households of different sizes by taking households with family size less than or equal to 4 and greater than 4 as the samples, respectively. The quality of human capital is reflected in rural households in terms of age and education accumulation. Household human capital follows the law of life cycle, with the growth of age, health and cognitive level of the differences in the level of human capital accumulation of the elderly is lower than the level of young people; the level of education level of the farm household directly affects its cognitive level and thus constrains the choice of livelihood strategies, but also determines the differential impact of their fiscal expenditure on the governance of relative poverty. Therefore, the differences in the response of households to fiscal support by age of the head of household are discussed with the distinction of the sample mean of 52. Differences in the policy response of households with different levels of education are discussed in terms of whether or not they have completed compulsory education. The analysis of differences in household characteristics for poverty reduction performance is shown in Table 5.

**Table 5:** Results of tests of differences in household characteristics.

Variable	Model 10	Model 11	Model 12	Model 13	Model 14	Model 15	Model 16	Model 17
**Zone = 1**	**Zone = 0**	**Familysize ≤ 4**	***Familysize* > 4**	**Age ≤ 52**	***Age* > 52**	**Edu ≤ 9**	**Edu > 9**
***Finrate***	-0.511 * (0.266)	-0.635 * (0.377)	-0.586** (0.272)	-0.751*** (0.287)	-0.695*** (0.257)	-0.465 * (0.251)	-0.695** (0.287)	-0.594** (0.256)
***Control***	Yes	Yes	Yes	Yes	Yes	Yes	Yes	Yes
***Constant***	0.496*** (0. 161)	0.501** (0. 239)	0.621*** (0. 150)	0.711*** (0. 176)	0.779*** (0. 150)	0.386*** (0. 134)	0.651*** (0. 144)	0.629*** (0. 178)
***Time***	Yes	Yes	Yes	Yes	Yes	Yes	Yes	Yes
***Provin***	Yes	Yes	Yes	Yes	Yes	Yes	Yes	Yes
***Obs***	3383	6127	6034	3976	5058	4952	8727	1283
***Confidence interval***	90%	90%	95%	95%	95%	90%	95%	95%

Note:

* p < 0.1,

** p < 0.05,

*** p < 0.01 at 90% confidence intervals, same below.

Model 10 and Model 11 show the regression results of the samples from the eastern and central and western regions, respectively. From the test results, the sample coefficient of the eastern coastal region is -0.511, while the coefficient of the independent variable in the central and western regions is -0.635, which are both significant, that is, the policy response of farmers in the eastern coastal region is lower than that in the central and western regions. The reason for this is that the eastern coastal region has a high level of economic development, and the needs of farm households are characterised by the improvement of the quality of life and the satisfaction of their basic public services or tenure. The sources of income are diversified, the level of non-farm income is high, and the degree of policy dependence is low. Moreover, with the saturation of fiscal expenditure scale efficiency in the eastern region, further promotion of fiscal poverty reduction faces greater marginal costs, and the marginal performance of fiscal poverty reduction decreases. Compared with the high level of non-farm development in rural areas in the eastern region, the construction and economic level of rural areas in the central and western regions are still lagging behind, and the impetus for rural self-development is still insufficient, and there is a parasitic urban-rural relationship under the intervention of government forces and the passive involvement of peasants, with higher levels of reliance on and responsiveness to policies[16].

Household size reflects the developmental endowment of the household to a certain extent, comparing Model 12 with Model 13, large family farmers have a higher level of response to the policy, with a coefficient on the independent variable of -0.751, which is significantly larger than that of -0.586 in Model 12. Large families have a concentrated demand, better information and resource sharing, richer social capital accumulation, and relatively abundant labour force, so they are more responsive to the fiscal support policy; and with the increase in the number of working people in the family, the relaxation of income constraints allows family members to have better choices of livelihood strategies, and to flexibly adjust their livelihood strategies in the face of the fiscal support policy.

As the head of household grows older, households in the life cycle tend to age to a certain extent, and lack flexibility and innovation in their response to policy changes and in the implementation of household decisions[57], and the health of family members is gradually declining, so the management of multidimensional relative poverty is relatively difficult. In contrast, younger-headed households show greater adaptability and flexibility in responding to policy changes, and are better able to take advantage of the opportunities presented by policy. This corresponds to the findings of Models 14 and 15, i.e., the regression coefficient of the independent variable in Model 14 is -0.695, which is greater than the -0.465 in Model 15.

From Models 16 and 17, The regression coefficients of Model 16 were significantly higher than those of Model 17,the marginal performance of fiscal support to agriculture is lower for farmers with high education accumulation than for those with low education accumulation, which is contrary to the findings of most studies. However, in the case of multidimensional relative poverty governance, it is not only about the improvement of a single dimension of income. Households headed by households with low levels of education, whose needs lie in the fulfilment of basic needs and whose development potential and endowments are lacking, respond to policy provision with a focus on direct economic subsidies and support, resulting in a high level of policy dependence and a high degree of responsiveness; Whereas households with high levels of education focus on opportunities for economic development, have better development potential and endowments, and have sufficient incentives for self-growth, resulting in a lower level of policy dependence. At the same time, the pro-poor and inclusive nature of macro-policies has led to the phenomenon of group selection in policy implementation, so that the low-education group is more vulnerable to the impact of policies.

### 4.4. Mediation effects test

The above has already proved that the input of fiscal support to agriculture can effectively improve the multidimensional relative poverty of farm households to achieve common prosperity, while the differences in household characteristics affect the performance of the policy, the article further needs to clarify how the fiscal support to agriculture can achieve the governance of the multidimensional relative poverty of farm households. Existing theories emphasise the role of capital to the countryside represented by digital financial inclusion, while playing the role of endogenous resources of social capital, so the article discusses the possible paths of these two. Because the article has a large sample size and a hierarchical structure, python is used to calculate and estimate the mediating effects of the structural equations, and the results are shown in Table 6.

**Table 6:** Results of the mediation effect test.

Variable	Model 18	Model 19	Variable	Model 20	Model 21
	*Lindex*	*Mpiscor*		*Lsocial*	*Mpiscor*
***Lindex***		0.019^**^ (0.008)	***Lsocial***		-0.0105^***^ (0.000)
***Finrate***	1.643^***^ (0.122)	-0.731^***^ (0.117)	***Finrate***	5.170^***^ (0.009)	-0.655^***^ (0.000)
***Constant***	-5.403^***^ (0.161)	0.557^***^ (0.069)	***Constant***	20.21^***^ (0.000)	0.680^***^ (0.000)
***Control***	Yes	yes	***Control***	yes	yes
***Direct Effect***	-0.7306^***^		***Direct Effect***	-0.6552^***^	
***Indirect Effect***	0.0317 ^*^		***Indirect Effect***	-0.0542^***^	
***Aggregate Effect***	-0.6989^***^		***Aggregate Effect***	-0.7095^***^	
***Obs***	10010	10010	***Obs***	10010	10010

Models 18-19 report the regression results with digital financial inclusion index as the mediating variable, while Models 20-21 report the regression results with social capital as the mediating variable. From Model 18, fiscal support to agriculture can significantly increase the level of digital financial inclusion, but from Model 19, the effect of digital financial inclusion on multidimensional relative poverty is significantly positive, i.e., there is a masking effect. The results of the mediation effects show that the direct effect is significantly negative, while the indirect effect is positive, implying that the role of digital financial inclusion has some ‘pull’ on the governance of fiscal multidimensional relative poverty in the current period. Although the overall poverty reduction performance of fiscal support to agriculture exists and contributes to digital financial inclusion, it is undeniable that digital financial inclusion is not conducive to the advancement of poverty reduction to a certain extent. The reason may lie in the short-term negative spillover effects of digital financial inclusion, which are manifested in the mismatch of financial resources and the lack of financial literacy of farm households[58]. Digital financial inclusion resources tend to be concentrated in areas with higher economic bases and tend to be invested in low-risk and more accessible customers, while the use of financial instruments, which likewise demand financial literacy from farm households, will exacerbate multidimensional relative poverty by excluding disadvantaged groups. Thus hypothesis H2a is tested.

The mediating role of social capital and hypothesis H3a are also confirmed in the regression findings. The regression coefficient of ‘Expenditure on favours’ as a proxy variable for social capital in Model 21 is significantly negative for the dependent variable. In the mediation effect results, the total effect -0.7095, direct effect -0.6552, indirect effect -0.0542, all significant. Fiscal inputs for agriculture significantly increase the social capital of farm households and further alleviate the multidimensional relative poverty of farm households. Fiscal inputs and project support encourage community co-operation and collective collaboration and promote the accumulation of social capital among farm households. Cooperation among heads of households, information sharing and resource integration within villages have effectively improved the living conditions of farming households; The expansion of social networks and the building of social trust have supported the development of agricultural production, improved the productivity of farming households and promoted the realization of common wealth.

### 4.5. Further analysis

The above conclusion that digital financial inclusion constrains the governance of multidimensional relative poverty in the current period, in terms of time, it is necessary to discuss whether there is a long-term exacerbation of multidimensional relative poverty by digital financial inclusion, so the article constructs a dynamic panel model with a first-order lagged variable of digital financial inclusion, which is incorporated into the model to discuss the time-lagged effect. In addition, in the theoretical analysis, it is considered that social capital has the potential effect of inhibiting common wealth, so the quadratic term of social capital is constructed to discuss its non-linear relationship. The results are presented in Table 7.

**Table 7:** Results of further analyses.

Variable	Model 22	Variable	Model 23
	*Mpiscor*		*Mpiscor*
***L.Mpiscop***	0.124^***^ (0.023)		
***Lindex***	0.465^***^ (0.155)	***Lsocial***	0.009^***^ (0.000)
***L.Lindex***	-0.280^***^ (0.087)	***Lsocial*** ^***2***^	-0.0022^***^ (0.000)
***Finrate***	-0.300 ^*^ (0.089)	***Finrate***	-0.674^***^ (0.241)
***Constant***	-0.494 (0.369)	***Constant***	0.695^***^ (0.132)
***Control***	Yes	***Control***	Yes
***Obs***	8008	***Obs***	10010

Model 22 reports the results of the dynamic panel regression analysis of the level of digital financial inclusion, which shows that the role of digital financial inclusion plays a lagging role, and the coefficient of the first-order lag term is -0.280, which is significantly negative at the 95% confidence level. Over time, households can ‘learn’ to adapt to the existence of digital financial inclusion as a tool and provide positive feedback. In addition, financial resources can be disseminated among households through social networks, thereby benefiting all households and achieving multidimensional relative poverty management, which implies to some extent that social capital as an informal system has a synergistic effect on the formal system of financial inclusive development. Model 23 reports the nonlinear effect of social capital on multidimensional relative poverty, with the primary term of 0.009, significantly positive at the 95% confidence level, and the estimated coefficient of the secondary term of -0.0022, significantly negative, suggesting that an increase in social capital will exacerbate multidimensional relative poverty at lower levels of social capital, and that the governance of multidimensional relative poverty can be realised only when social capital exceeds a certain level. When the social capital is too low, the family social network is lacking, the family has the possibility of being marginalised, easy to lead to information asymmetry and the lack of mutual aid mechanisms, while the same period of time rich in social capital of the family is prone to “elite capture”, so exacerbating the multidimensional relative poverty[59]. Only when the threshold is crossed, cooperation and mutual aid in the village increases, information mobility improves, and social trust is strengthened, so as to facilitate better use of resources and opportunities by farm households, and thus achieve effective governance of multidimensional relative poverty. Therefore, in areas with weak social capital development, policies should focus on both institution building and transparency in resource allocation to avoid resources being controlled by a few. In summary, hypotheses H2b and H3b are verified.

The uneven level of economic development and diverse cultural composition of Chinese society make it necessary to explore the differentiating characteristics of “elite capture,” as shown in Table 8. Clans, formed on the basis of bloodline, were a major part of Chinese people’s lives and an important organizational basis before the modern era. Since the Ming and Qing dynasties, wars and government-led migrations have led to the formation of atomized societies in the north. Although clans existed in the north, the process of clan growth was frequently interrupted, making large-scale settlement difficult [60]. The South, on the other hand, has formed stable and large clans in a relatively stable social environment and closed geography, and has a direct impact on today’s grassroots social governance; for this reason, the article discusses the North-South differences in the elite capture phenomenon separately. Table 8 Model 24 reports the nonlinear regression results of social capital in the southern provinces, while Model 25 shows the estimation results in the northern provinces. Comparing the regression coefficients of social capital, the coefficients of the primary and secondary terms in the southern provinces are 0.014 and -0.0023, respectively, which are significantly larger than those in the northern provinces, indicating that the nonlinear role of social capital is more prominent in regions with a thriving clan culture and the strength of informal institutions, and that social capital will exacerbate social inequality in extreme cases. Under the rural clan tradition, there is a high degree of familiarity among farm households, and there is a strong spatial boundary and a tendency to maintain a rigid class structure in the countryside, which accentuates inequality in the case of extreme social capital [30]. It can also be seen that in the southern region, the coefficient of the government’s fiscal support to agriculture in the context of considering the nonlinear role of social capital is -0.250, which is negative but insignificant, and the estimated value is smaller than the sample of the northern region in Model 25. It suggests that the role of the formal government system will be weakened when social capital is at the extremes.

**Table 8:** Expression of Heterogeneity in Elite Capture.

Variable	Model 24	Model 25	Model 26	Model 27	Model 28	Model 29
	*Sourth*	*North*	*Coastal*	*Inland*	*Old*	*Young*
***Lsocial***	0.014^***^ (0.003)	0.007^***^ (0.002)	0.009^**^ (0.003)	0.009^**^ (0.003)	0.011^***^ (0.002)	0.012^**^ (0.003)
***Lsocial*** ^***2***^	-0.0023^***^ (0.000)	-0.0021^***^ (0.000)	-0.0019^***^ (0.000)	-0.0022^***^ (0.000)	-0.0023^***^ (0.000)	-0.0019^***^ (0.000)
***Finrate***	-0.250 (0.252)	-0.969^***^ (0.210)	-0.532 ^*^ (0.266)	-0.633 (0.326)	-0.458 ^*^ (0.215)	-0.716^**^ (0.246)
***Constant***	0.499 (0.014)	0.684^***^ (0.170)	0.590^***^ (0.149)	0.535 ^*^ (0.223)	0.489^***^ (0.124)	0.804^***^ (0.138)
***Control***	Yes	Yes	Yes	Yes	Yes	Yes
***Obs***	2559	7451	3883	6127	4952	5058

China’s economic development since modern times has greatly changed the original social structure and state of mind, and at the same time, it has greatly impacted the rural social structure and social capital that is bound by blood ties. In addition, with the deepening of economic construction, government power is more deeply embedded in the grassroots, curbing inequality at the grassroots level. The economically developed coastal areas in eastern China, with their high level of marketization, have been accompanied by improvements in government grassroots governance capacity and the embedding of formal institutions. The formation and promotion of Zhejiang Province’s “Fengqiao Experience” and the introduction of the “Thousand Villages Renovation, Ten Thousand Villages Demonstration” project in 2024 are the best examples. To verify this view, Model 26 discusses the nonlinear role of rural social capital with a sample of eastern coastal areas, and Model 27 discusses the nonlinear role of social capital with a sample of non-coastal areas. From the results, the coefficient of the quadratic term of social capital is significant and lower than that of the non-coastal region in the coastal region with a high level of economic development, and the coefficient of fiscal support for agriculture is also significantly negative. It indicates that the increase in the level of marketization will impact on the original closed human social structure and improve the nonlinear role of social capital.

Since the reform and opening up, rural social change has been accompanied by a shift in the mode of rural household division of labor, and more young family members have begun to be deeply embedded in the non-farming society. Model 28 and Model 29 consider the differences in sample regression results under different age levels of household heads, respectively, with classification criteria consistent with the heterogeneity analysis above. The estimated coefficient of the quadratic term in Model 28 for samples with household heads older than 52 years old is -0.0023, which is significantly negative and larger than the coefficient of -0.0019 for samples with low-age household heads. It suggests that older rural household groups are more likely to fall into inequality conditional on uneven social capital. Younger household groups tend to have a higher level of education and are deeply influenced by the market economy, so that agricultural production and rural life are no longer their main productive life, but more part-time or non-agricultural production. For this reason, their social capital goes beyond the scope of geography and blood ties, and is more centered on business ties. The diversified composition of social capital makes them less vulnerable to the extremes of rural social capital distribution, and government policies are more effective in helping them.

In summary, the nonlinear characteristics of social capital are influenced by historical factors, cultural traditions, social structure and other factors, with significant regional characteristics. The non-linear characteristics of social capital also constrain the performance of the formal system’s fiscal poverty alleviation, so poverty alleviation policies should be formulated differently according to local conditions, with full consideration of local traditions and realities.

## 5. Conclusions and recommendations

Focusing on multidimensional relative poverty is a requirement for consolidating the results of poverty eradication and improving the quality of poverty governance. Based on provincial and household microdata, the article empirically examines the role and influence mechanism of fiscal support to agriculture on the multidimensional relative poverty of farm households. The study shows that: the input of fiscal support to agriculture can effectively alleviate the multidimensional relative poverty of farm households; In terms of the intensity of policy response, farm households in the central and western regions, with larger, younger and less educated families have higher intensity of policy response, and such households have flexible adjustability of livelihood strategies or higher dependence on policies; The level of digital financial inclusion is developed in rural areas under the guidance of fiscal funds, and the level of financial inclusion in rural areas. However, this cannot play a positive role in alleviating multidimensional relative poverty in the current period, and the positive financial spillover has a time-lag effect; The social capital of rural households will be strengthened and greatly improve the multidimensional relative poverty of rural households under the input of fiscal funds, but the improvement of social capital on relative poverty has a non-linear effect, and the lack of social capital in the governance of villages is prone to inducing the “elite capture” and exacerbating group differences. And the “elite capture” of social capital is characterized by significant regional and family variability, resulting from historical traditions, market shocks and changes in family structure. Based on the above conclusions, the article makes the following recommendations to better realise the common prosperity of farmers and improve the quality of poverty governance:

First, fiscal policies and expenditures should be strengthened and made more effective and precise. Focusing on the function of fiscal as the “foundation and important pillar of national governance”, fiscal funds should be invested more in weak areas and links. We will optimise the mechanism for allocating fiscal resources to support agriculture, tilting them towards rural areas in the central and western regions, and towards rural infrastructure construction, education, healthcare and other public services, so as to continuously improve the level of rural infrastructure and public services. Improve the precision, fairness and transparency of fiscal capital investment. Using big data technology, relative poverty groups will be precisely targeted, households will be accurately targeted through “object-oriented” management, and the use of funds will be adjusted in a timely manner through dynamic monitoring. Strengthening grass-roots governance capacity, ensuring that fiscal funds are properly utilised, and improving the mechanisms for supervising the use of fiscal funds and evaluating their performance, so as to ensure the efficiency of their use.

Secondly, the coordination of various policies should be strengthened with a systemic concept. We are highlighting the role of fiscal policy as an “incentive guide”, giving full play to the synergistic effect of policies, providing good policy-based financial support, and guiding financial capital to the countryside in an orderly manner. Improve the construction of financial infrastructure in rural areas, enhance the accessibility of financial services, and continue to broaden the breadth and depth of rural financial capital services. Do a good job of financial business publicity and training, improve the accumulation of knowledge among rural households, and reduce the policy lag in positive incentives for financial services. Strengthen financial service supervision, dynamically assess the performance of financial capital relative to poverty governance, and adjust financial support strategies in due course.

Thirdly, attention should be paid to the cultivation of social capital and the avoidance of low-level “elite capture”. The Government should support the development of rural communities, guide farmers to participate in collective economic organizations, such as cooperatives and women’s groups, so as to achieve the integration and expansion of farmers’ social networks; Continuously advancing the construction of rural cultural and ethical standards, utilizing modern multimedia technology for educational promotion, promoting traditional virtues, improving village regulations and agreements, advocating for new norms of civility, and fostering social trust and norms among rural households to enhance their social capital. This will maximize the positive role of social capital in reducing relative poverty.Explore the goal of building a “tipping point” for social capital, based on the actual situation of each region, and explore internal and external resources to guide social capital to reach the optimal level of marginal effect. Improve villagers’ autonomy and build a fair, transparent, clean and efficient grass-roots governance platform, so as to prevent the over-concentration and uneven distribution of resources and avoid “elite capture”.

Fourthly, synergise the roles of the formal and informal systems. The integration of formal fiscal and financial instruments with informal social capital resources should be improved, and the mutual assistance and cooperation mechanisms provided by social capital should be used to strengthen the credit endorsement of financial services and increase the accessibility of financial capital. Use social networks to build a platform for information and knowledge transfer, promote the spread of digital technology and alleviate information asymmetry. Relying on social norms and social trust, strengthen the regulation of financial services and curb the negative spillover effects of inclusive finance.

## 6. Research limitations and challenges to the implementation of policy recommendations

Although the article clearly demonstrates the impact of fiscal inputs on the alleviation of multidimensional relative poverty in rural China, and innovatively recognizes the time-lag effect of digital financial inclusion and the phenomenon of “elite capture” of social capital in the path of implementation, the article still has room for further insights into the availability of fiscal support at the household level, and the variability of the type and structure of social capital, due to the limitations of data availability. However, the article is limited by the availability of data, and there is still room for further in-depth research on the availability of fiscal support and differences in the types and structure of social capital at the household level, which needs to be supported by a wider range of public data sources.

Accordingly, the article puts forward policy recommendations on the synergies between fiscal inputs, financial support, and social capital. However, in the context of China’s economic slowdown and the fiscal pressure brought by the COVID-19 epidemic, the sustainability of fiscal inputs has become a major challenge for policy implementation, while the administrative efficiency, integrity, and resource allocation capacity of the grassroots government have a direct impact on the effectiveness and performance of fiscal funds. Although inclusive financial instruments have provided farmers with greater access to financial services, the smallholder group generally suffers from slow capital accumulation and insufficient credit, which makes the financial supply unable to meet their needs and exacerbates the lagging effect of financial support in the poverty reduction process. The cultivation of social capital is a long and gradual process, especially in some clan-based rural areas and communities where informal authority is too strong. The rigidity of grass-roots governance structures and the phenomenon of elite capture make it difficult for social capital to be distributed equitably, which makes it difficult for poor groups to benefit from the accumulation of social capital. With the gradual transformation of the traditional rural social structure, the effective cultivation of social capital still faces greater challenges. The potential constraints posed by the above factors on the implementation of fiscal poverty reduction policies also provide practical reference value on how to carry out effective policy implementation.
