# Correction: Deeper effects of fiscal multidimensional poverty reduction: Household characteristics, financial lags and elite capture

**DOI:** 10.1371/journal.pone.0322317

**Published:** 2025-04-04

**Authors:** Wenjie Jiang, Hong Yang, Chunyu Liu

The authors, Wenjie Jiang, Hong Yang, Chunyu Liu are incorrectly noted as co-corresponding authors. The correct corresponding author is Chunyu Liu. Chunyu Liu’s email address is: 505377140@qq.com.

## References

[1] Jiang W, Yang H, Liu C. Deeper Effects of fiscal multidimensional poverty reduction: household characteristics, financial lags and elite capture. PLoS One. 2025;20(2): e0319255. doi: 10.1371/journal.pone.0319255 39992927 PMC11849867

